# Crystal structure of human cyclin-dependent kinase-2 complex with MK2 inhibitor TEI-I01800: insight into the selectivity

**DOI:** 10.1107/S0909049513020736

**Published:** 2013-09-26

**Authors:** Aiko Fujino, Kei Fukushima, Takaharu Kubota, Tomomi Kosugi, Midori Takimoto-Kamimura

**Affiliations:** a Teijin Institute for Bio-medical Research, Teijin Pharma Limited, 4-3-2 Asahigaoka, Hino-shi, Tokyo 191-8512, Japan

**Keywords:** MK2, MAPKAP-K2, CDK2, X-ray, structure, inhibitor

## Abstract

Mitogen-activated protein kinase-activated protein kinase 2 (MK2 or MAPKAP-K2) is a Ser/Thr kinase from the p38 mitogen-activated protein kinase signalling pathway and plays an important role in inflammatory diseases. The crystal structure of the MK2–TEI-I01800 complex has been reported; its Gly-rich loop was found to form an α-helix, not a β-sheet as has been observed for other Ser/Thr kinases. TEI-I01800 is 177-fold selective against MK2 compared with CDK2; in order to understand the inhibitory mechanism of TEI-I01800, the cyclin-dependent kinase 2 (CDK2) complex structure with TEI-I01800 was determined at 2.0 Å resolution. Interestingly, the Gly-rich loop of CDK2 formed a β-sheet that was different from that of MK2. In MK2, TEI-I01800 changed the secondary structure of the Gly-rich loop from a β-sheet to an α-helix by collision between Leu70 and a *p*-ethoxyphenyl group at the 7-position and bound to MK2. However, for CDK2, TEI-I01800 bound to CDK2 without this structural change and lost the interaction with the substituent at the 7-position. In summary, the results of this study suggest that the reason for the selectivity of TEI-I01800 is the favourable conformation of TEI-I01800 itself, making it suitable for binding to the α-form MK2.

## Abstract 2

The Gly-rich loop of cyclin-dependent kinase 2 (CDK2) bound to TEI-I01800 as an MK2 specific inhibitor forms a β-sheet which is a common structure in CDK2–ligand complexes. Here, the reason why TEI-I01800 does not become a strong inhibitor against CDK2 based on the conformation of TEI-I01800 is presented.

## 1. Introduction

Mitogen-activated protein kinase-activated protein kinase 2 (MK2 or MAPKAP-K2) is one of the Ser/Thr kinases from the p38 mitogen-activated protein kinase signalling pathway, which has been shown to play an important role in the production of TNF-α and other cytokines (Beyaert *et al.*, 1996 ▶). TNF-α is implicated in several inflammatory diseases such as rheumatoid arthritis, and therefore inhibition of TNF-α activity represents a most promising target for anti-inflammatory therapy (Camussi & Lupia, 1998 ▶; Kotlyarov *et al.*, 1999 ▶). Several groups have reported programs to develop anti-inflammatory therapies through the generation of MK2 inhibitors and determined the crystal structure of MK2 (Wu *et al.*, 2007 ▶; Hillig *et al.*, 2007 ▶; Velcicky *et al.*, 2010 ▶; Anderson *et al.*, 2007 ▶, 2009*a* ▶,*b* ▶; Revesz *et al.*, 2010 ▶; Argiriadi *et al.*, 2009 ▶, 2010 ▶; Fujino *et al.*, 2010 ▶; Barf *et al.*, 2011 ▶). With the exception of two inhibitor complex structures, TEI-I01800 [Protein Data Bank (PDB) code 3a2c; Fujino *et al.*, 2010 ▶] and 2,4-diaminopyrimidine derivative from Abott (PDB code 3ka0; Argiriadi *et al.*, 2010 ▶), all MK2 complexes deposited in the PDB have a β-sheet Gly-rich loop (β-form). In a previous study (Fujino *et al.*, 2010 ▶) we revealed that the MK2 complex with TEI-I01800 has a unique α-helical Gly-rich loop (α-form) and TEI-I01800 binds to a specific hydrophobic pocket exposed by the structural change.

Protein kinases are key regulators of cell function that constitute one of the largest and most functionally diverse gene families. Kinases are particularly prominent in signal transduction and coordination of complex functions. In particular, cyclin-dependent kinase 2 (CDK2), which is also a member of the Ser/Thr kinase family, plays a central role in the control of the cell cycle (Tsai *et al.*, 1991 ▶) and interference with the cell cycle *via* inhibition likely to be an undesirable feature in anti-inflammatory drugs used chronically such as MK2 inhibitor; in fact, the improvement of selectivity over CDK2 has been published (Anderson *et al.*, 2009*a* ▶,*b* ▶; Kosugi *et al.*, 2012 ▶). MK2 and CDK2 complex structures with the same inhibitor which has only 29-fold selectivity against MK2 are reported and the interaction mode is observed to look similar (Anderson *et al.*, 2009*a* ▶). These structures show that it is difficult to obtain selectivity for MK2 by interaction with the hinge region.

TEI-I01800 shows a good potency and selectivity against significant kinases and has 177-fold selectivity for MK2 over CDK2. In this study we present a CDK2–TEI-I01800 complex structure for the purpose of better understanding the binding mode and the structure guided drug design. Consequently, the Gly-rich loop of CDK2 keeps the β-form and TEI-I01800 binds to CDK2 with an unfavourable conformation compared with the stable conformer TEI-I01800 itself. The results indicate that TEI-I01800 is a specific compound which causes the structural change of the Gly-rich loop of MK2, not CDK2, to increase selectivity for MK2.

## 2. Materials and methods

Inactive monomeric human CDK2 was expressed in Tn5 insect cells using a recombinant baculovirus encoding CDK2 gene and purified as described in the literature with slight modification (Rosenblatt *et al.*, 1993 ▶). The purified CDK2 was concentrated to 5–10 mg ml^−1^ and crystallization experiments of apo-CDK2 were performed using the sitting-drop vapour-diffusion method under the conditions of 0.1 *M* HEPES pH 7.4, 10–15% PEG 3350 and 50 m*M* ammonium acetate. After the crystals were grown for 2–3 d, TEI-I01800, which was synthesized by Kosugi *et al.* (2012 ▶), was added to the crystallization drop at a final concentration of 2 m*M* and soaked for 12–24 h. X-ray diffraction data were collected on beamline BL32B2 at SPring-8 at 100 K using 25% glycerol as cryo-protectant. The CDK2–TEI-I01800 complex crystals diffracted to 2.0 Å resolution and belonged to space group *P*2_1_2_1_2_1_ with unit-cell parameters *a* = 53.64, *b* = 72.10 and *c* = 72.61 Å. The reflection data were processed using *CrystalClear* 1.3.5 (Rigaku) and molecular replacement was carried out using *MOLREP* (Vagin & Teplyakov, 1997 ▶) from CCP4 (Collaborative Computational Project, 1994 ▶) with the CDK2 structure (PDB code 1fvt) without ligand and waters as the initial model. Structure refinement was carried out using the program *REFMAC* (Murshudov *et al.*, 1997 ▶). After rigid-body refinement, the electron density for TEI-I01800 was clearly found and constructed using *COOT* (Emsley & Cowtan, 2004 ▶). The structure refinement was continued until the *R* and *R*_free_ factors were 18.9% and 24.9%, respectively. Statistics of the data collection and final structure are summarized in Table 1 ▶. Methods for measurement of MK2 and CDK2 enzyme assay were described in our previous paper (Kosugi *et al.*, 2012 ▶). Structure minimization of the protein–ligand complex with OPLS 2005 force field was performed using the *Protein Preparation Wizard*. Conformational search and potential energy calculation was carried out using *Macromodel* in the Schrödinger software suite (http://www.schrodinger.com/). All figures were produced using *Discovery Studio* (Accelrys; http://accelrys.com/).

## 3. Results and discussion

### 3.1. Overall structure of CDK2–TEI-I01800 and the binding mode of TEI-I01800

**Figure 1:**
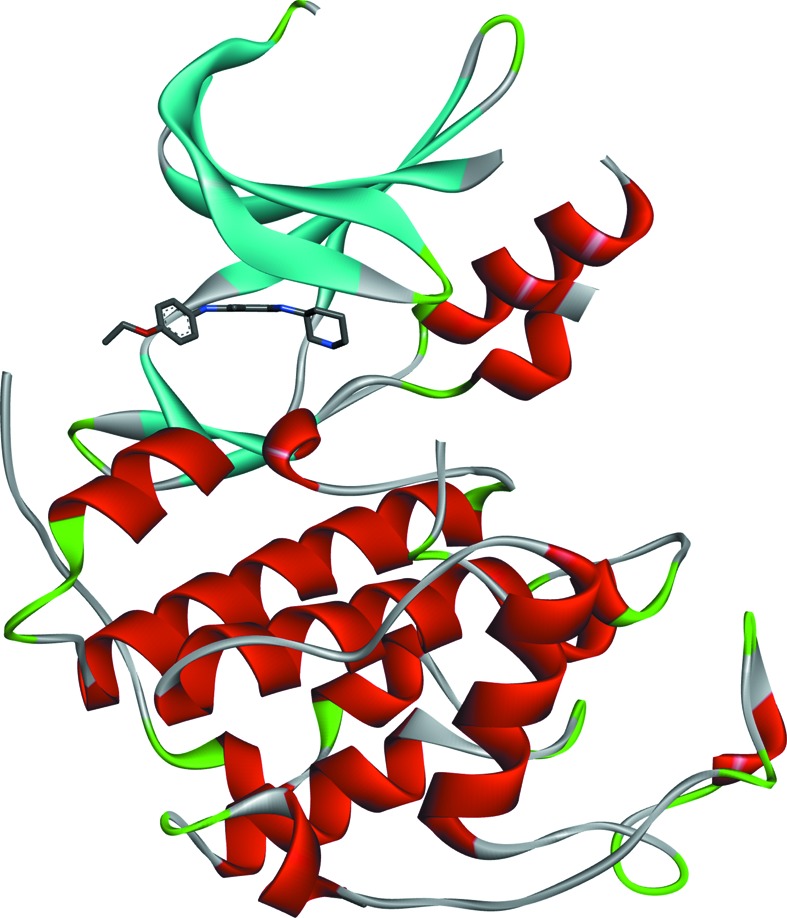
Overall structure of the CDK2–TEI-I1800 complex.

The overall structure of the CDK2–TEI-I01800 complex is very similar to that of other CDK2 structures. The density maps from Val154 to Glu162 (T-loop) are disordered. In spite of its weak inhibition, the electron density map of TEI-I01800 is clearly found in the ATP-binding pocket at the N-terminal domain, and TEI-I01800 binds to CDK2 with several hydrogen-bond interactions (Figs. 1 ▶ and 2 ▶).

The molecular structure and atomic numbering of TEI-I01800 are shown in Fig. 3 ▶. It has a pyrazolo[1,5-*a*]pyrimidine scaffold with a (3*S*)-piperidylamino group at the 5-position, a methyl group at the 6-position and a *p*-ethoxyphenylamino group at the 7-position. Its inhibitory activity for CDK2 is 23 µ*M* and for MK2 is 0.13 µ*M* (IC_50_).

**Figure 2:**
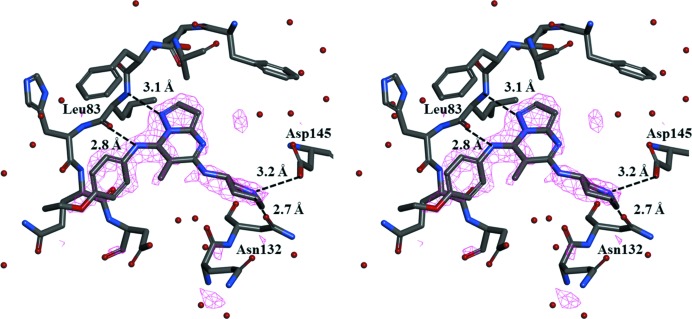
Stereodiagram of the electron density map of TEI-I01800 (*F*_o_ − *F*_c_ OMIT map contoured at 3.0 σ) and binding interactions.

**Figure 3:**
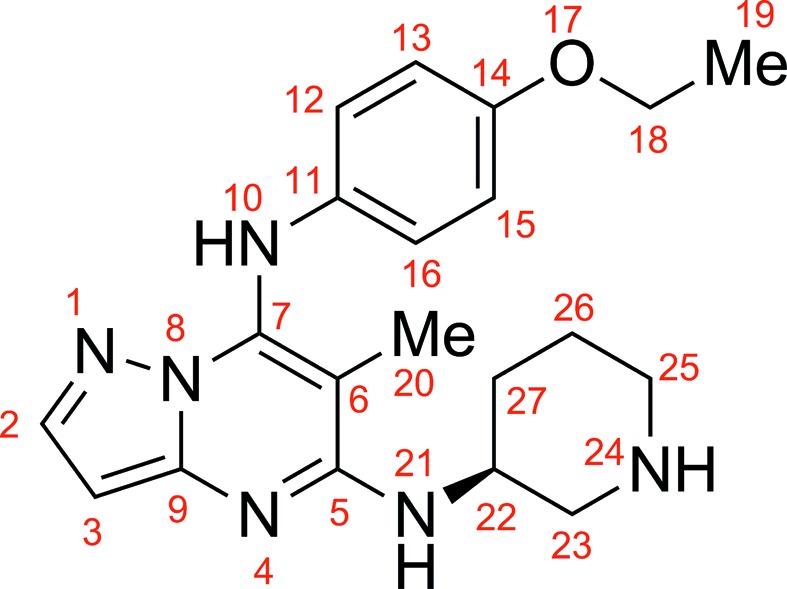
Schematic molecular structure of TEI-I01800.

As shown in Fig. 2 ▶, TEI-I01800 interacts with the backbone amide of Leu83 and through two hydrogen bonds: TEI-I01800 N1—Leu83 N (3.1 Å) and TEI-I01800 N10—Leu83 O (2.8 Å). The N24 atom of TEI-I01800 must be ionized by hydrogen bonding to the carboxyl group of Asp145 (3.2 Å). This atom also makes one additional hydrogen bond to the OD1 atom of Asn132 (2.7 Å). TEI-I01800 is twisted around two freely rotatable bonds (C7—N10 and N10—C11) and the *p*-ethoxyphenylamino group at the 7-position is slightly twisted toward the C-terminal domain to avoid steric hindrance.

### 3.2. Structure comparison with other CDK2 structures

At present, 335 structures of human CDK2 are available from the PDB, 237 of them are inactive monomeric CDK2, and almost all are complexes with small inhibitors for drug design. The others are active heteromers with several kinds of cyclin molecules; they show the large conformational change of the ^45^PSTAIRE^51^ helix, the T-loop and the Gly-rich loop (Jeffrey *et al.*, 1995 ▶). In our study the inactive CDK2 was selected to develop the selective MK2 inhibitor, because the SAR (structure activity relationship) for this series is correlated with X-ray structure analysis.

**Figure 4:**
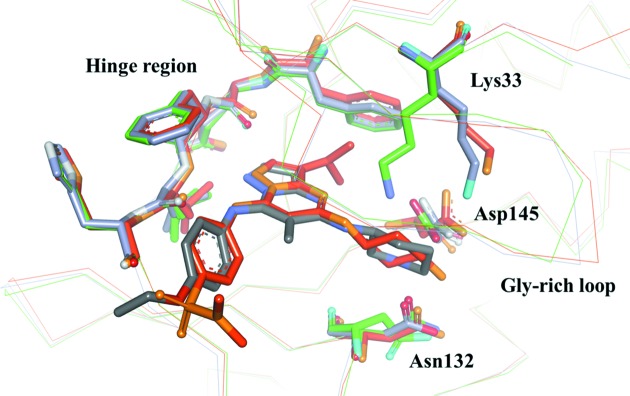
Superposing of the CDK2–TEI-I01800 (grey) on the apo-CDK2 (green) and the CDK2–pyrazolo[1,5-*a*]pyrimidine inhibitor from Vernalis (orange).

The Gly-rich loop of CDK2 is considered flexible because it moves by the binding of the inhibitor or cyclin. However, all CDK2 with the exception of CDK2/cyclinA/peptide inhibitor p27KipK (Russo *et al.*, 1996 ▶) keep the β-form and the secondary structural change as of MK2–TEI-I01800 is not found. This suggests that the β-form is stable in CDK2–small inhibitor complexes and the structural change to the α-form does not occur. Fig. 4 ▶ shows the superimposition of CDK2–TEI-I01800 on the highest-resolution apo-CDK2 (PDB code 4ek3; Kang & Stuckey, 2013 ▶) and a pyrazolo[1,5-*a*]pyrimidine inhibitor which has the same scaffold as TEI-I01800 from the Vernalis complex (PDB code 1y91; Williamson *et al.*, 2005 ▶). The root-mean-square deviation (RMSD) between Cα atoms is 0.83 and 0.55 Å, respectively. Although the overall structure is almost the same including the Gly-rich loop structure, there are two differences found between apo-CDK2 and complexes. One is Lys33 and Asp145, and the other is the lack of part of a T-loop. In apo-CDK2, Lys33 makes a salt bridge with Asp145, but, in complex, Asp145 contacts with the ligand amine, and Lys33–Asp145 bonding is broken. As described in the literature (Wu *et al.*, 2003 ▶), the movement of Lys33, Asp145 and adjacent Tyr15 results in an induced movement of the Gly-rich loop and rearrangement adjacent to the T-loop. The disorder of the T-loop found in CDK2–inhibitor may be caused by the movement of Lys33 and Asp145. The binding conformation and interaction of the CDK2–Vernalis compound is very similar to that of TEI-I01800, except for the interaction between Phe80 and an isopropyl moiety at the 3-position. Because our study also showed that this area is a promising region for CDK2 activity, we focused on positions other than the 3-position. Although we expected the dihedral angle between the pyrazolopyrimidine scaffold and the 7-position moiety of TEI-I01800 to be large based on the result of the MK2–TEI-I01800 analysis, no significant difference was shown in comparison with the Vernalis compound which is unsubstituted at the 6-position.

### 3.3. Conformational analysis of TEI-I01800

**Figure 5:**
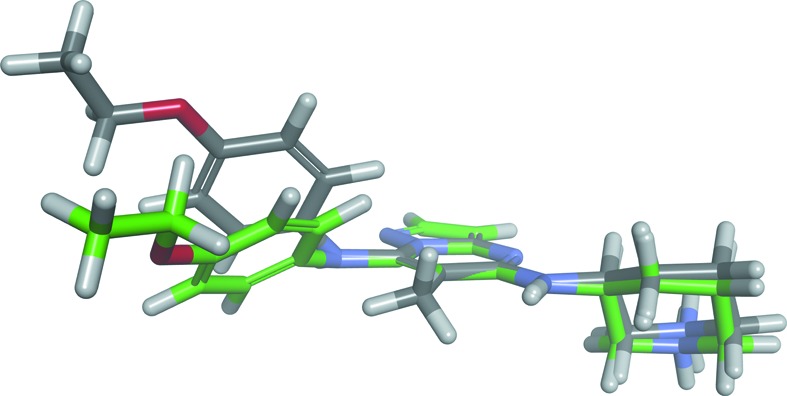
Stable conformers of TEI-I01800 (grey) and TEI-I01800 without the methyl group at the 6-position (green).

As described in our previous paper (Kosugi *et al.*, 2012 ▶), the selectivity of TEI-I01800 derivatives was increased by methylation at the 6-position. In order to understand the effect of the methyl group at the 6-position, con­formational analyses were performed. The ideal dihedral angle of TEI-I01800 between the 7-position substituent and the pyrazolo[1,5-*a*]pyrimidine scaffold (C6–C7–N10–C11) is calculated to be 58.4° because the 7-*p*-ethoxyphenyl group and the 6-methyl group are twisted by the steric hindrance. When the 6-methyl group is removed, the calculated dihedral angle decreases to 18.8° and the position of the 7-*p*-ethoxyphenyl group is clearly different as shown in Fig. 5 ▶. This analysis strongly supports our SAR data that the non-planar conformation of TEI-I01800 is important for MK2 inhibition. On the other hand, the observed dihedral angle of TEI-I01800 binding to CDK2 is smaller (−29.2°) than that of MK2 (44.9°), which is measured after structural idealization by the energy minimization, and the potential energy is calculated as −120.7 kcal mol^−1^ and −309.8 kcal mol^−1^, respectively. The result indicates that the planar conformation of TEI-I01800 as found in CDK2 is unstable compared with the non-planar conformation of TEI-I01800 found in the MK2 complex.

### 3.4. Structure comparison with MK2–TEI-I01800 and selectivity of TEI-I01800

CDK2 was superimposed on MK2 by the least-squares method between main-chain atoms against the residues Phe80–Leu83 (Met138–Leu141 in MK2), Asn132 and Asp145 (Asn191 and Asp207 in MK2); the RMSD value calculated for these six residues is 0.80 Å and that for 180 residues which are aligned by a superimposed structure is 2.93 Å. The ATP-binding sites with the exception of the Gly-rich loop are well overlapped. The two hydrogen-bonding interactions, as in MK2, between the hinge region and TEI-I01800 are slightly stronger than that of the MK2–TEI-I01800 complex (TEI-I01800 N1—Leu141 N is 3.1 Å and TEI-I01800 N10—Leu141 O is 3.3 Å); this result is consistent with other reports (Anderson *et al.*, 2009*a* ▶). However, the number of hydrogen bonds between CDK2 and the 5-(3*S*)-piperidylamino group is less than for MK2 which has an additional hydrogen bond (TEI-I01800 N10—Glu190 O; 2.8 Å). The lacking hydrogen bond is one factor of the selectivity, but our result indicates a more interesting mechanism.

**Figure 6:**
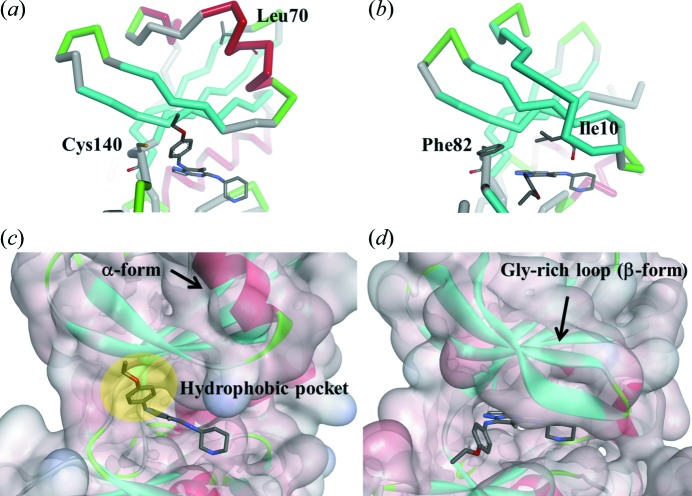
ATP-binding pocket of (*a*), (*c*) MK2 and (*b*), (*d*) CDK2 bound to TEI-I01800.

The ATP-binding pockets of MK2 and CDK2 with TEI-I01800 are shown in Fig. 6 ▶. In MK2–TEI-I0800 the Gly-rich loop flips up toward the N-terminal domain by the collision between Leu70 and TEI-I01800 and forms the α-helical Gly-rich loop. Then, this structural change induces the hydrophobic pocket for the binding of the *p*-ethoxyphenyl group at the 7-position. Because the optimization of the 7-position clearly showed an improvement in the MK2 activity, the interaction between this pocket and TEI-I01800 is considered important (Kosugi *et al.*, 2012 ▶). On the other hand, CDK2 cannot be induced to an α-form by TEI-I01800 although Ile10 which corresponds to Leu70 in MK2 has the possibility to be a trigger of such a structural change. Two reasons why CDK2 does not undergo the structural change as MK2 are considered. One is that the β-form of CDK2 is more stable than the α-form compared with MK2 because, although a huge number of monomeric inactive CDK2 complex structures with various inhibitors have been reported in the PDB, there is no α-form CDK2. The other reason is that Phe82 in CDK2 is larger than Cys140 in MK2 and could cause a second collision between Phe82 and a stable conformer of TEI-I01800 binding to MK2 (CE1—C17 is 1.9 Å and CE1—C20 is 2.0 Å). As a result, CDK2 cannot take the α-form, and the binding pocket, which is not favourable for binding with the stable conformer of TEI-I01800, has a distinct shape compared with that of MK2.

As described above, because the stable conformer of TEI-I01800 matches the binding conformation of TEI-I01800 in MK2, TEI-I01800 can stably bind to MK2, which has the possibility of causing a structural change of the Gly-rich loop. However, when TEI-I01800 binds to the β-form of CDK2, the dihedral angle between the 7-position substituent and the scaffold must be arranged in an unfavourable conformation. In conclusion, the results indicate that the characteristic α-form structure of MK2 suitable for TEI-I01800 binding and the favourable interaction different from that of CDK2 is the main reason for the selectivity of TEI-I01800.

## 4. Conclusion

In this research we present the CDK2 structure bound to potent and selective MK2 inhibitor TEI-I01800. Although TEI-I01800 creates the α-form MK2 by collision with Leu70, in CDK2 TEI-I01800 does not change the Gly-rich loop structure. Instead, the conformation of TEI-I01800 itself is arranged so that it is possible to interact with the β-form CDK2. TEI-I01800, which is a non-planar structure due to the steric repulsion between the 6-methyl group and the 7-*p*-ethoxyphenyl group, cannot keep the stable non-planar conformation in the narrow ATP-binding pocket in the β-form CDK2. Thus, it binds with an unstable conformation and also loses the important interaction with the *p*-ethoxyphenyl substituent at the 7-position for MK2 inhibitory activity. We conclude that this difference is the reason TEI-I01800 demonstrates the selectivity for MK2 and not CDK2.

In the development of kinase inhibitors, since the shape of the ATP-binding pocket is quite similar, it is very difficult to have selectivity for off-target kinases. As this study has shown, the selective inhibitor can change the protein structure to arrange the shape of the pocket suitable for binding by the compound itself. The mechanism of such an induced fit means that it will become possible to acquire a compound with higher selectivity.

## Supplementary Material

PDB reference: 3wbl

## Figures and Tables

**Table 1:** Data-collection and refinement statistics Values in parentheses are for the highest-resolution shell.

Data collection	
Beamline	SPring-8 BL32B2
Wavelength (Å)	1.000
Resolution (Å)	50.00–2.00 (2.07–2.00)
Mosaicity (°)	0.30
No. of unique reflections	19590
*R*_merge_ (%)	5.9 (20.4)
Completeness (%)	99.9 (100.0)
Multiplicity	7.03 (7.23)
Average *I*/σ(*I*)	18.5 (8.4)
Space group	*P*2_1_2_1_2_1_
Unit-cell parameters (Å)	
*a*	53.64
*b*	72.10
*c*	72.61

Refinement	
Resolution (Å)	20.0–2.0
*R* factor (%)	18.9
*R*_free_ (%)	24.9
No. of reflections (work/test)	18564/1001
RMSD from ideal values	
Bond lengths (Å)	0.018
Bond angles (°)	1.64
